# What can we learn from corporate sustainability reporting? Deriving propositions for research and practice from over 9,500 corporate sustainability reports published between 1999 and 2015 using topic modelling technique

**DOI:** 10.1371/journal.pone.0174807

**Published:** 2017-04-12

**Authors:** Nadine Székely, Jan vom Brocke

**Affiliations:** Institute of Information Systems, University of Liechtenstein, Vaduz, Principality of Liechtenstein; Indian Institute of Technology Madras, INDIA

## Abstract

Organizations are increasingly using sustainability reports to inform their stakeholders and the public about their sustainability practices. We apply topic modelling to 9,514 sustainability reports published between 1999 and 2015 in order to identify common topics and, thus, the most common practices described in these reports. In particular, we identify forty-two topics that reflect sustainability and focus on the coverage and trends of economic, environmental, and social sustainability topics. Among the first to analyse such a large amount of data on organizations’ sustainability reporting, the paper serves as an example of how to apply natural language processing as a strategy of inquiry in sustainability research. The paper also derives from the data analysis ten propositions for future research and practice that are of immediate value for organizations and researchers.

## Introduction

Growing legislative pressure and increasing public concern about the global climate and the carrying capacity of the earth have led to increasing demands for organizations to act in sustainable ways [1]. Consequently, the number of organizations that publish information on their sustainability practices has grown steadily [2]. One way in which organizations communicate these practices to stakeholders is through sustainability reports—usually published annually with financial reports [3]—that report on the organization’s “economic, environmental and social impacts caused by its everyday activities” [4].

For the last fifteen years, researchers have sought to shed light on the publication of organizational sustainability practices in sustainability reports in order to determine how organizations interpret the challenge of sustainability. Some researchers have focused on the frequency of reporting and other high-level information in order to gain insights into the general development of sustainability reporting [2,5], while others have used qualitative content analysis techniques to provide an overview of certain organizations’ reporting practices [1]. Other research has examined the content of sustainability reports in a more quantitative way through text-mining techniques, focusing in particular on the frequency of certain terms that are related to sustainability practices [1,3,6,7]. Another study explored the references made to ecological limit by analysing the context of use of a predefined list of terms related to ecological limit [8]. Besides the last study, all of these studies have taken only a limited number of reports into consideration.

In contrast, the present study employs text-mining techniques to conduct topic modelling on 9,514 sustainability reports published between 1999 and 2015. In particular, we apply Latent Dirichlet Allocation (LDA), which is used to identify themes and their distribution in large collections of documents [9].

We extend the current research on sustainability reports in three ways. First, we use a recent data sets by including sustainability reports that were published as recently as the beginning of 2015, but we also extend the time frame back to 1999. Second, we analyse a significantly large number of sustainability reports—9,514 reports. By extending the time frame and the number of reports, we include a high diversity of reports in terms of sector and published year, which allows us to show the development of topics over time and their distribution among sectors. Third, to our best knowledge, we apply a methodology—LDA—that has not yet been used to examine sustainability reports. This method allows us to examine the documents without a predefined list of terms and thus provides us with a broader view on the content of the sustainability reports than other studies were able to gain.

In seeking to shed light on organizations’ sustainability reporting, we focus on identifying (1) sustainability practices and their development over time; (2) the coverage of economic, environmental, and social aspects in sustainability reports; and (3) the differences in sustainability reporting (and practices) among certain sectors [3].

The paper proceeds as follows. The next section provides background on corporate sustainability and sustainability reports. Then we describe the data and methods used. After presenting and discussing the results of our analysis, we conclude in the last section.

## Research background

### Corporate sustainability

Through the Brundtland Commission’s publication of the report, *Our Common Future*, the concept of sustainability and particularly the definition of sustainable development as “meet(ing) the needs of the present without compromising the ability of future generations to meet their own needs” [10] has gained popularity [11]. The term *corporate sustainability* is often used in the context of organizations, but it has no commonly accepted definition [11]. Some authors focus on the environmental aspects of sustainability, others on the social aspects, and others take an integrated view, combining sustainability’s environmental, social, and economic aspects without prioritizing any one dimension [11–13]. We see corporate sustainability as lying at the interface of economic contribution, environmental performance, and social responsibility [14]. Further, we agree with the work of Dyllick and Hockerts [15], that these three dimensions of corporate sustainability can be seen distinct on an operational level, but should be integrated on strategic level.

Definitions of sustainable practices differ among the sectors in which organizations operate, but sustainable practices can generally be divided into environmental, social, and economical sustainable practices. The environmental practices refer to the consumption of natural resources and the release of emissions, both of which should be below a rate that ensures the health of the eco-system [15]. Thus, the environmental practices are concerned with reducing environmental degradation through the conservation of resources, including energy [16], and sustainable waste management [17]. Reporting on the environmental practices focuses on eco-control, environmental cost accounting, and life cycle analysis [17,18]. Potential indicators of environmental performance are air emissions, biodiversity, energy use, noise, resource depletion, solid waste, transport, and water use and discharge [14].

The social practices aim at adding value to the local community [15] and helping to maintain stable communities and their quality of life [16] under the umbrella of human rights [17]. Besides corporate citizenship, corporate philanthropy [17], social partnership, and social sponsorship [14], the social practices focus on the development of human capital [17] through, for example, employee training programs, improvement management, apprentice programs, fringe benefits, flexible work time models, health and prevention programs, flexible workplace design, qualification programs for job returnees, minority-promotion programs, and occupational child care [18]. Other topics include stakeholder involvement and customer satisfaction [14].

The economic sustainability practices reflect the guarantee of long-term liquidity and above-average return to the stakeholders [15]. These practices include corporate governance, risk and crisis management, codes of conduct and compliance, corruption and bribery, talent attraction and retention [17], promotion of economic viability [16], economic profitability, and economic equity [19].

Organizations are driven to engage in sustainability by regulatory pressures [14,20,21], pressure from customers and employees [21], and pressure from the organization’s management, as investment in sustainable practices may improve financial performance [22]. In order to implement sustainability in an organization, it must be ensured that sustainability is embedded in the overall strategy, that it has organizational support (including top-management support, bottom-up support), that it includes all business units, that stakeholders are intrinsically and extrinsically motivated, and that the sustainability performance data can be tracked [23]. Barriers to the adoption of sustainable practices include concerns about the ease of implementation and production risks [24].

One way that organizations’ sustainability activities can become visible is through the publication of corporate sustainability reports [11]. Here, we review the history of sustainability reports and the corresponding research on these reports.

### Sustainability reports

Organizations’ reporting of non-financial data started in the 1970s with “*social balance sheets*” [25]. At first, organizations reported on the social benefits they paid to their employees quantitatively [25]. Later they also included information on product quality and social engagement [25]. After several environmental catastrophes in the 1980s, organizations started to report on the environmental aspects of their efforts as well [25], with the first publication of a separate environmental report in 1989 [2]. In the following years, the focus shifted solidly to environmental reports and from somewhat argumentative reporting to proactive reporting with competitive elements [25]. Consequently, today’s sustainability reports are often seen as marketing instruments. Involvement of public relations departments and third parties in the compilation of sustainability reports, as well as industry-specific foci because of industries’ differing stakeholders, also suggest that sustainability reports are often used as marketing instruments [1,26]. Around 2000, the focus shifted again to include more social and financial aspects of companies’ sustainability efforts [1–3,25]. While in 1999, 98 percent of the reports published by the largest 250 multinationals were concerned only with environmental issues, by 2002 this percentage had declined to 71 percent [2]. In addition, the names of the reports changed from *corporate citizenship report* (emphasizing the social aspect), to *corporate (social) responsibility report* and then finally to *sustainability report* [25]. The number of organizations that report on their sustainability activities has steadily increased to the point at which sustainability reports have become standard procedure [1]. Today many reports follow the format published by the Global Reporting Initiative (GRI) [2], but even though the reports increasingly focus on performance indicators, improvement in the creditability of these figures is needed, as organizations often report only a few indicators, sometimes provide only summarized figures, and do not indicate whether the figures are estimates or measures or how changes were made [1]. Many organizations follow the GRI standard to increase the credibility of their reports [27], and stakeholders are often directly involved in determining the content of the reports [28].

The number of companies that publish sustainability reports differs from sector to sector [2]. In the past, in industrial sectors like chemicals, computers and electronics, cars, utilities, oil and gas, and food and beverages, the number of organizations that publish sustainability reports was higher than average [2], while financial companies, trade and retail, services, communications, and media were less active in reporting their sustainability activities [2]. Since the amount of companies publishing a sustainability report for the first time is decreasing since 2003 [1], one might expect that this distribution among sectors might still be true today.

## Method

We employ a semi-automated text-mining technique on publicly available sustainability reports to determine the topics they address. These techniques usually represent documents as vectors. In the easiest form, such a vector includes for each term in the document the number of appearance. However, such a vector has a high number of dimensions (each one reflecting one term). Thus, we need to reduce the dimensionality of the resulting vector [29] in order to be able to handle these huge amount of data.

For this, we are using LDA since, in the resulting vector of LDA, each dimension corresponds to one topic or concept [29]. A topic is a probability distribution over all of the terms that co-occur in the underlying documents [29] and one document is a probability distribution itself over all topics in the corpus [30,31]. That means, when describing a topic, the author takes words with a certain probability from the pool of terms related to that topic [31]. For instance, when writing about the topic of climate change, terms like climate, CO_2_, emissions, GHG, warming, or temperature have a high probability of appearing, while terms like employee benefits, social responsibility, or gains have a lower probability.

Topics are identified through considering which terms are often occurring together, thus, it is assumed that the more often terms occur in the same document, the more likely it is, that they belong to the same topic. Each sustainability report consists of several topics. The probability distribution of one of these documents shows how prominent the identified topics are in this specific report.

### Pre-processing

Before the analysis begins, a few data pre-processing steps are necessary: (1) collecting data, (2) converting documents from pdf to text files, (3) filtering out documents that are not in English (to avoid issues related to translation), (4) tokenizing (splitting the documents into words [32]), (5) cleaning the text, (6) lemmatizing, and (7), removing stop words.

We collected 15,351 sustainability reports published between 1999 and 2015 for our study. We retrieved the PDF documents from the GRI website (http://database.globalreporting.org/search) by crawling and scraping the contents automatically. For each document, we collected metadata like publication year, company, and sector. With the help of a Python script we created, we converted these pdf files into text files for further analysis. In the course of these steps, we excluded 1,152 encrypted documents that could not be extracted easily. To avoid issues related to translation, we limited the documents to those written in English, so we used another Python script to identify those documents. The remaining 9,514 sustainability reports written in English serve as the basis for all further analyses.

Next, we tokenized the documents—split them into tokens that include words and special symbols like punctuation marks—and cleaned up the text by bringing all characters into lower-case and removing special characters and numbers. Then we lemmatized the words using the WordNetLemmatizer, and eliminated standard stop words (i.e., general-purpose words like articles, pronouns, and conjunctions). For this, we used the stop word list “English” provided by the NLTK package of Python. Further, we removed terms that appeared in fewer than two documents. We manually checked the remaining vocabulary to exclude other irrelevant terms like country names.

### Latent Dirichlet Allocation (LDA)

The purpose of the LDA process is to find in each document a mix of topics, where each topic is described by a mix of terms [31]. Thus, the probability distribution of the mix of topics differ from that of the mix of terms [31]. The hyper-parameter α describes the shape of the per-document topic distribution, and the hyper-parameter β describes the shape of the per-topic word distribution [32]. The distributions are estimated by the algorithm using Dirichlet priors [31]. Gensim (for Python) and Mallet (for Java) are among the extant efficient and effective implementations of LDA [29].

We use the Mallet implementation, which automatically estimates the hyper-parameters α and β, to conduct the LDA analysis. The number of topics, defined in advance, depends on the intended level of topic specialization [31]. We wanted to assign labels to each of the resulting topics, but for when there are too few dimensions, the topics tend to be more general, as they are a broad mixture of terms that makes it difficult to assign specific labels, and when there are too many, the topics become too specific. We employed the algorithm on three, five, ten, twenty, fifty, seventy, and one hundred dimensions and compared the results, deciding to focus on seventy dimensions, as this number gave us a broad variety of topics without going into too much detail.

The algorithm produced two result sets per topic. The first result set consists of all terms of the corpus and the degree to which they are likely contribute to the topic [31]. The second result set contains all documents in the corpus and the probability that the corresponding topic occurs in the document.

In interpreting the results, the five to twenty most probable terms for each topic are usually examined in order to identify the degree of commonality and, thus, specify the label of the topic [29]. Our analysis focused on the terms with the twenty highest probabilities. Five researchers, including the first author of this work, examined the seventy topics and classified each as describing environmental sustainability, social sustainability, economic sustainability, sustainability in general, or no sustainability at all. All researchers were provided with definitions of environmental, social, and economic sustainability based on the practices described in the research background of this paper. Since sustainability reports also contain information that is not related to sustainability, we expected to find several topics that were not relevant to our further analysis.

In the next step, we continued the examination of the topics that are relevant to sustainability by analysing the prominence of industries in each topic in terms of their mean probability of occurring in the topic. We excluded a few topics that consisted of terms that seemed to describe sustainability practices but instead described the business of the most probable sectors. For instance, one topic contained words like *oil*, *gas*, and *energy*, which appear to describe energy sources as a topic of environmental sustainability. However, analysis of the most probable sectors showed that this topic is used primarily by the energy sector, so it probably describes their business activities. Therefore, we excluded this topic. For the remaining topics, we analysed the mean probability per year, per country, per continent, and per organization size. We found all information except that for the continent in the meta-data provided by the GRI database and assigned countries to their continents based on a map from the United Nations Statistics Division (http://unstats.un.org/unsd/methods/m49/m49regin.htm).

We also assigned to each topic that was relevant to sustainability a label that describes the topic’s content. Therefore, the first author of this study made one proposal based on the twenty most probable terms of each topic, discussed it with the second author, and resolved any disagreement. Thereby, labels were selected in order to represent the twenty most probable terms (those terms that are used with high probability when describing the topic) of the specific topic.

Table 1 provides an overview on the conducted steps as well as the main decisions that had to be made in order to conduct the analysis.

**Table 1:** Overview on LDA process.

Main steps performed	Main decisions made
1. Pre-Processing (Preparation of documents) a. Collecting data b. Converting documents from pdf to text files c. Filtering out documents that are not in English d. Tokenizing e. Cleaning the text f. Lemmatizing g. Removing stop words	1. Selection of data source 2. Selection of stop word (terms that are excluded from further analysis)
2. Latent Dirichlet Allocation analysis	3. Selection of algorithms 4. Number of topics
3. Interpretation	5. Assigning topics to sustainability dimensions (environmental, social, economic, general, and not related to sustainability) 6. Identification of relevant topics (related to sustainability, not industry-specific) 7. Labelling of relevant topics

## Results

We analyse 9,514 sustainability reports published between 1999 and 2015 by 3,906 different organizations. The most common industries were financial services, followed by the energy sector, the mining sector, and food and beverage products.

We find forty-two topics that are related to sustainability. We conduct several analyses for each topic, including its development over time, its distribution over industries, countries, continents, and size of organization. Based on this analysis, we come up with ten observations that are summarized in Table 2 and are described in the following. Further, the Appendix contains an overview of all seventy topics, including the most prominent terms of each one as well as the probability of occurrence (how high is the chance that the specific term appears in the context of this topic) of these terms in the context of this topic.

**Table 2:** Results of analysis.

#	Observation
1	Organizations report on environmental, social, and economic sustainability.
2	Topics on environmental, social, and economic sustainability are equally distributed.
3	Economic sustainability topics are of increasing importance for organizations.
4	As to environmental sustainability, organizations report on emissions and energy consumption.
5	Biodiversity and renewable energy sources receive little attention in reports by organizations.
6	Regarding social sustainability, organizations report on labour practices.
7	Customer orientation is in organizations’ focus.
8	Sponsorship activities for social sustainability focus on schools and education.
9	Economic sustainability reporting is based on financial data.
10	Sustainability actions are both general and context-specific in nature.

### Observation 1: Organizations report on environmental, social, and economic sustainability

During our first interpretation phase, we looked at each topic/collection of terms and assigned this topic to environmental sustainability, social sustainability, economic sustainability, general sustainability or not related to sustainability. The corresponding assignment can be found in the table in Appendix. We can find topics that are related to environmental sustainability, as well as topics that are related to social or economic sustainability. Thus, we state that organizations report on environmental, social, and economic sustainability.

### Observation 2: Topics on environmental, social, and economic sustainability are equally distributed

In total, we identify 42 topics that are related to sustainability from which we assigned 31 topics to be either related to environmental or social or economic sustainability. In total, there are eight topics related to environmental sustainability, 13 topics related to social sustainability and eleven topics related to economic sustainability. Thus, all three dimensions are covered by roughly the same number of topics.

### Observation 3: Economic sustainability topics are of increasing importance for organizations

**Fig 1:**
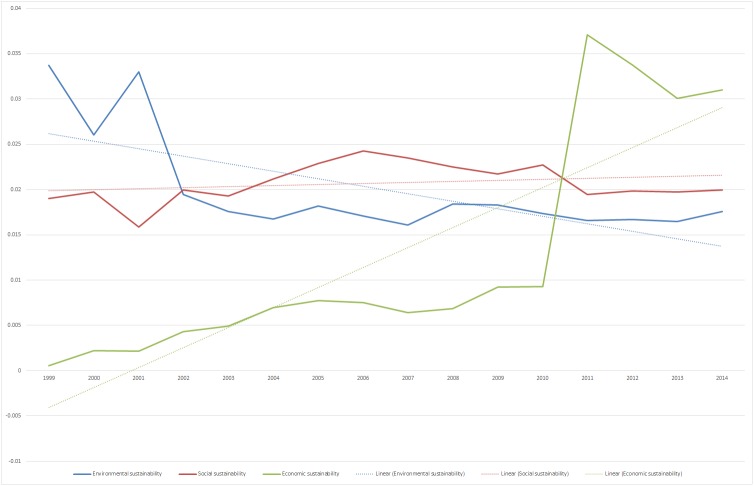
Trend analysis of mean probability of occurrence of environmental, social, and economic topics in the years 1999 until 2014.

For each topic, the LDA algorithm provides us with the probability that this topic appears in a specific document. And for each document we know the year in which it was published. In order to understand how the probability of a topic changed over years, we calculated the mean probability for this topic in all documents of a specific year. We further calculated this mean probability not only for one specific topic but for a group of topics, e.g., all topics that we previously assigned to being related to economic sustainability. Fig 1 provides an overview on the development of the mean probabilities of each of the three dimensions. As the linear trend line shows, the probability of environmental sustainability topics is slightly decreasing, while the one of social sustainability topics is more or less stable. The trend line of economic topics shows a constant increase. Particularly, the probability that an economic topic appeared in a sustainability report strongly increased from 2010 to 2011. Thus, we observe that economic sustainability topics are of increasing importance for organizations.

### Observation 4: As to environmental sustainability, organizations report on emissions and energy consumption

We find eight topics that are related to environmental sustainability. Two topics (no. 16 and no. 32) refer to environmental sustainability performance and environmental sustainability data respectively. Four topics are concerned with environmental sustainability in the supply chain. Topic no. 10 (green supplier) focuses on the supplier as part of the global supply chain. Environment-related terms are *energy* and *emissions*. Topic no. 21 (production) and topic no. 45 (green production) are, of course, concerned with production. Among the most probable occurring terms are *safety*, *emissions*, and *fuel* in topic no 21 and *material*, *energy*, *waste*, *reduction*, *recycling*, *emissions*, and *impact* in topic no. 45. Topic no. 35 (production and packaging) broadens the focus by including packaging, particularly recycling. Most probable occurring terms for this topic are *recycling*, *waste*, *water*, *safety*, and *health*. Two topics focus on environmental sustainability in certain contexts: Topic no. 53 summarizes terms from building construction, and environmental-sustainability-related terms are *energy*, *green*, *sustainable*, *environmental*, *water*, and *material*. Topic no. 63 summarizes terms that relate to water management, including wastewater treatment.

Table 3 provides an overview on all these topics, including the most probable terms for each topic. The most probable terms are those terms that have the highest probability (noted as percentage after each term) to appear if a text is about the specific topic.

**Table 3:** Topics related to environmental sustainability.

Topic No.	Label	Most probable terms
10	**Green supplier**	supplier 1.81%; product 1.45%; employee 1.19%; technology 1.12%; global 1.05%; chain 1.00%; supply 0.98%; **energy 0.91%;** data 0.89%; program 0.74%; **emission 0.64%;** solution 0.60%; information 0.54%; business 0.51%; system 0.46%; content 0.45%; management 0.42%; material 0.42%; world 0.40%; human 0.40%
16	**Environmental sustainability performance**	percent 2.17%; program 1.92%; **energy 1.59%**; facility 1.07%; goal 0.77%; community 0.72%; state 0.69%; **emission 0.68%;** environmental 0.66%; organization 0.53%; effort 0.52%; employee 0.50%; water 0.49%; gas 0.47%; center 0.44%; ton 0.44%; system 0.43%; reduce 0.40%; metric 0.40%
21	**Production**	product 2.44%; plant 2.23%; chemical 2.15%; cement 1.98%; material 1.87%; production 1.33%; safety 1.30%; **emission 1.26%;** site 1.08%; industry 0.87%; raw 0.80%; business 0.74%; process 0.70%; million 0.68%;; el 0.66%; customer 0.65%; concrete 0.60%;industrial 0.54%; dow 0.51%; ton 0.48%
32	**Environmental sustainability data**	**emission 2.38%;** sustainability 2.08%; reporting 1.90%; performance 1.82%; data 1.72%; gri 1.64%; total 1.56%; **energy 1.40%;** environmental 1.17%; indicator 1.12%; waste 1.04%; employee 1.04%; consumption 1.03%; scope 0.96%; management 0.90%; stakeholder 0.89%; material 0.84%; assurance 0.83%; impact 0.76%; number 0.72%
35	**Production & Packaging**	product 4.12%; packaging 2.19%; consumer 1.97%; water 1.61%; material 1.26%; business 1.06%; brand 1.04%; waste 1.03%; supplier 0.88%; beverage 0.81%; manufacturing 0.75%; plant 0.67%; safety 0.62%; recycling 0.58%; health 0.57%; production 0.56%; raw 0.56%; life 0.51%; plastic 0.51%; chain 0.48%
45	**Green production**	product 4.34%; environmental 2.83%; production 1.45%; system 1.26%; material 1.25%; **energy 1.24%;** manufacturing 1.11%; waste 0.99%; technology 0.93%; customer 0.85%; site 0.80%; reduction 0.73%; plant 0.70%; recycling 0.69%; **emission 0.68%;** substance 0.63%; process 0.63%; supplier 0.62%; sale 0.62%; impact 0.62%
53	**Building construction**	construction 4.09%; building 4.00%; project 3.99%; **energy 1.66%**; development 1.57%; green 1.41%; design 1.24%; site 1.21%; office 0.92%; work 0.86%; housing 0.81%; land 0.79%; home 0.75%; engineering 0.73%; sustainable 0.71%; environmental 0.70%; award 0.70%; water 0.66%; area 0.66%; material 0.57%
63	**Water management**	water 9.53%; waste 3.26%; plant 2.18%; environmental 1.48%; treatment 1.24%; management 0.95%; system 0.93%; service 0.85%; area 0.83%; environment 0.81%; quality 0.80%; wastewater 0.80%; total 0.76%; supply 0.72%; **energy 0.64%;** project 0.57%; local 0.49%; work 0.49%; resource 0.48%; **emission 0.43%**

For most of the environmental sustainability related topics, the terms energy and emissions as well as related terms such as gas, ton, waste, or consumption are among the twenty most probable terms, showing that organizations focus on energy consumption and emissions (including waste) specifically.

### Observation 5: Biodiversity and renewable energy sources receive little attention in reports by organizations

While energy is well covered in the topics related to environmental sustainability, we could not find evidence that renewable energy was discussed in the reports as well. Further, the probability of the term biodiversity was close to zero, meaning that the chance of appearing in one of the sustainability reports is very low. Consequently, we conclude that biodiversity and renewable energy sources receive little attention in the analysed sustainability reports.

### Observation 6: Regarding social sustainability, organizations report on labour practices

We find thirteen topics that are related to social sustainability. Six of these topics are concerned with employees and labour practices, each has a unique focus. Table 4 shows the most probable terms of each of these topics. *Employee safety* and *work time* belong to the five topics with the highest mean probability over all analysed topics, which further show the relevance of these topics for organizations.

**Table 4:** Social sustainability topics concerned with employees including most probable occurring terms ordered by their probability.

Topic No.	Label	Most probable terms
**0**	**Employee safety**	Employee, safety, management, health, training, performance, risk, environmental, corporate, environment, community, system, operation, process, business, local, compliance, supplier, standard, ensure
**4**	**Employee diversity**	Program, employee, corporate, community, business, responsibility, global, organization, support, diversity, work, environmental, commitment, social, service, citizenship, effort, team, leadership, initiative
**5**	**Work time**	People, work, employee, time, team, make, day, life, event, child, part, family, working, good, office, member, staff, manager, care, environment
**19**	**Management**	Management, system, business, employee, activity, sustainability, social, program, green, global, KRW, support, growth, issue, performance, customer, overseas, stakeholder, information, partner
**26**	**Development program**	Programme, organisation development, center, group, social, staff, management, service, labor, training, system, department, sustainable, environmental, quality, responsibility, aim, work, policy
**43**	**Employee responsibility**	Employee, group, corporate, management, activity, responsibility, project, system, social, training, work, environmental, principle, product, total, environment, support, business, member, information

### Observation 7: Customer orientation is in organizations’ focus

**Fig 2:**
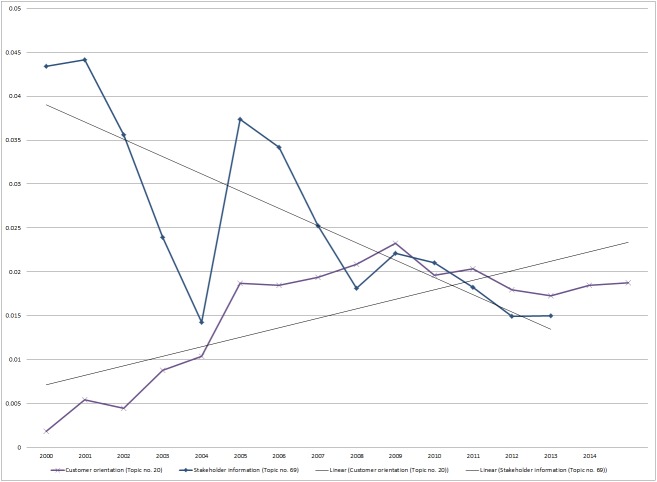
Trend analysis of mean probability of occurrence of topic no. 20 and no. 69 in the years 2000 until 2014.

Two topics related to social sustainability focus on stakeholder involvement. Topic no. 69 refers to stakeholder information and consists of probable terms like *program*, *community*, *organization*, *management*, *performance*, *government*, *work*, *people*, *information*, *reporting*, and *development*. Topic no. 20 (customer orientation) focuses on one specific stakeholder, the customer. Probable terms in this topic include *customer*, *service*, *product*, *responsibility*, *satisfaction*, *information*, *online*, and *survey*. Analysing the development of the mean probability of these both topics between 2000 and 2014, we find that the mean probability of topic no. 20 is slightly increasing over time while the trend for topic no. 69 shows a slight decrease (see Fig 2). To summarize, the customer is one only stakeholder that is mentioned in a separate topic and this topic is further of increasing prominence in the sustainability reports, showing the importance of this topic for organizations.

### Observation 8: Sponsorship activities for social sustainability focus on schools and education

One topic related to social sustainability (topic no. 60) specifically addresses sponsorship, more precisely, school sponsorship. Among the most probable terms are *school*, *project*, *education*, *child*, *development*, *support*, *foundation*, *initiative*, and *partnership*. After a peak in 2004, the probability of occurrence of this topic in a sustainability report remained more or less stable.

### Observation 9: Economic sustainability reporting is based on financial data

Of the ten topics related to economic sustainability, six are related to financial data. Table 5 shows the most probable terms for each of these financial data topics. Common probable terms are *share*, *board*, *risk*, *tax*, *consolidated*, *shareholder*, *asset*, *euro*, *cost*, and *loss*.

**Table 5:** Economic sustainability topics related to financial data including most probable occurring terms ordered by their probability.

Topic No.	Most probable terms
9	eur, group, euro, thousand, board, share, financial, market, asset, supervisory, member, general, consolidated, cost, activity, amount, shareholder, tax, dec, annual
18	share, group, financial, director, board, information, statement, document, meeting, million, consolidated, shareholder, capital, registration, asset, total, option, euro, plan, amount
29	group, share, board, annual, eur, total, financial, market, risk, million, member, sale, operation, pension, operating, change, liability, tax
39	financial, asset, statement, group, liability, cost, amount, loss, cash, fair, income, tax, note, risk, interest, recognized, rate, profit, impairment, annual
58	board, million, supervisory, delta, annual, share, result, member, van, management, Lloyd, statement, total, risk, policy, DSM, financial, remuneration, die
66	group, integrated, committee, risk, financial, management, business, board, performance, executive, annual, employee, director, share, remuneration, review, million, continued, operation, growth

**Fig 3:**
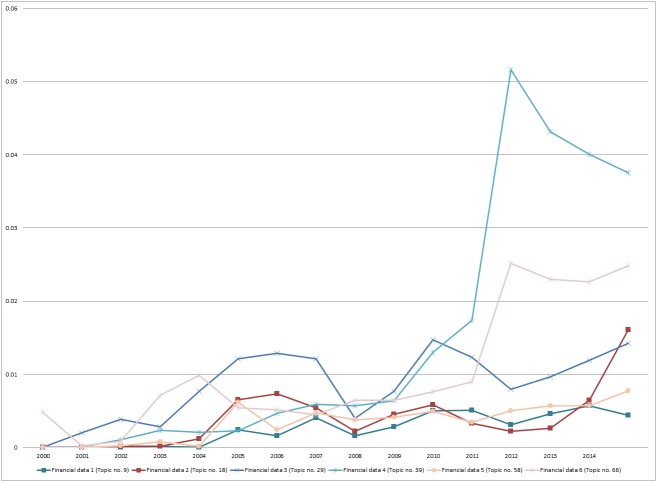
Trend analysis of mean probability of occurrence of financial data topics in the years 2000 until 2014.

The trend analysis of the probability of occurrence for these topics over time (Fig 3) shows that all financial data topics increase in probability over time, meaning that it is more likely that they are mentioned in sustainability reports.

### Observation 10: Sustainability actions are both general and context-specific in nature

For each topic, we also analyse how it is distributed over industries and countries respectively continents. That means, for each topic, we calculate the mean of the probabilities of all sustainability reports that were published by organizations that belonged to one specific industry or country. We gained the information about industry and country from the meta-data that we downloaded together with each sustainability report.

Many topics are kind of equally distributed over the available industries and countries, however, several topics are remarkable prominent on specific industries or countries. In the following, we only highlight those topics that differ from the average.

For the environmental sustainability topics, we find several prominent industries. For instance in the topics no. 10 (green supplier), 21 (production), and 35 (production & packaging) two industries are prominent: in topic 10, the computer industry and the technology hardware industry; in topic no. 21 the chemicals industry and the construction material industry; and in topic no. 35 the food and beverages industry and the households and personal products industry. In topic 45 (green production), the technology hardware industry, the consumer durables industry, and the equipment industry are the most prominent sectors. The construction and the real estate industries are concerned with *building construction*, while the water utilities industry and the waste management industry report about *water management*. Regarding the country, we find further significant differences between the two general environmental sustainability topics, as *environmental sustainability performance* is prominent mainly in North America, particularly in the United States. We also find differences among the supply chain topics. *Green supplier* is prominent in North America, while the production topics are most likely to occur in Asian reports. *Production and packaging* is most probable in Europe, followed by North America and Africa.

Analysing the topics related to social sustainability, topic no. 11 (social sustainability data) is particularly probable in the forest and paper products industry and in the agriculture industry, while the topic *work time* is most probable in reports from the toy industry. Topic no. 25 (sustainable development) is most probable in reports from Morocco or France. Topics no. 11 (social sustainability data) and no. 44 (corporate social responsibility) are most probable in the countries of South America; topic no. 11 is particularly common in Brazil. *Employee safety* is particularly prominent in North America and Asia, while *employee diversity* occurs primarily in the reports of multinational enterprises from North America. In Europe, *employee responsibility* is the most probable topic, while *management* is the most probable topic in reports from Asia. The topic *work time* is most probable in reports of small and medium-sized enterprises headquartered in Ecuador, while *stakeholder information* is more probable in small and-medium-sized enterprises in Australia and New Zealand.

The topics related to economic sustainability are the least focused on specific industries and countries. Only topics no. 39 (financial data 4) and no. 66 (financial data 6) are particularly probable in reports from Africa, as is *investment*.

We also find some topics that are related to sustainability in general. These topics are rather specific for certain industries and countries, for instance topic no. 30 (sustainability activities) is most probable in reports from the energy utilities sector, while *nuclear power* is, understandably, of particular interest to the energy industry. *Stakeholder issues* are mainly a topic of the tobacco industry. Topic no. 33 (CSR activities) is most probable in reports from the toys and the hardware technology industry. Topic no. 56 (development) is highly probable in reports from the mining industry. Further, topics no. 14 (organizational sustainability), no. 31 (sustainability program), and no. 33 (CSR activities) are most probable in reports from Asia. The probability of topic no. 30 (sustainability activities) is particularly high in Italian reports, while topic no. 41 (general sustainability) is more probable in reports from Oceania, and topic no. 50 (corporate sustainability) is more likely to appear in European reports. Sustainability projects are prominent only in Europe, and there particularly in Germany and Austria. The analysis of the companies’ nationality shows distinct results for the *nuclear power* topic, which is prominent mainly in Europe, but particularly in Belarus and the Russian Federation. *Stakeholder issues* is probable mainly in reports from Uganda, while the topic *annual meeting* has a high probability of appearing in reports from Africa, particularly South Africa and Namibia. Topic no. 56 (development) is most probable in reports from North America.

## Discussion

Our analysis applies topic modelling to more than 9,000 sustainability reports in order to identify sustainability practices. We identify forty-two topics that are related to sustainability from which we make ten observations. In the following, we discuss these observations and develop ten related recommendations for organizations and researchers.

### Observation 1: Organizations report on environmental, social, and economic sustainability

Coding the topics identified in the sustainability reports confirms the notion of the so-called triple bottom line [33], in that topics relate to environmental, social, and economic sustainability. From the forty-two topics, we assigned thirty-one to one of these dimensions. Even though the triple bottom line has been criticized for being difficult to implement [34], our results suggest that the three dimensions fit to structure organizations’ sustainability topics in practice, confirming previous results that organizations report on all of these dimensions [1–3,25]. The remaining eleven topics that were not assigned to one of the three dimensions are related to general sustainability topics that consist of a mix of terms that belong to all three dimensions, thus representing the integration aspect of the sustainability definition [35]. Hence, our results show that organizations report on the distinct dimensions of sustainability, but their reports also reflect the required integration of environmental, social, and economic sustainability. Particularly in the general sustainability topics, the focus seem to be more on strategic elements, for instance, terms like *business*, *group*, *and management* appear among the most probable terms. This would be in line with our understanding of sustainability that sustainability can be seen distinct on operational level, but should be seen integrated on strategic level. We recommend that organizations keep this distinction on their operational level but focus on integrating the three dimensions on a strategic level [15].

### Observation 2: Topics on environmental, social, and economic sustainability are equally distributed

While the common definitions of sustainability highlight the integration of its environmental, social, and economic dimensions [35], these dimensions have been seen historically as distinct. For instance, corporate sustainability origins are in environmental sustainability, while corporate social responsibility, which is today often synonymous with corporate sustainability, has its origins in social sustainability [12]. Before these two terms gained prominence, profit maximization and, therefore, economic value were seen as the core business functions [36]. Our study shows that the topics organizations report on can be more or less equally assigned to all three dimensions. Of the forty-two topics, slightly less than a quarter relates to environmental sustainability, slightly more than a quarter relates to social sustainability, and a quarter relates to economic sustainability. Thus, at least based on the number of topics, despite their origins, the three dimensions are nearly equally covered in the reports. However, this does not mean that the three dimensions are also equally covered in terms of depth or occurrence. Also previous studies found that organizations report on all three dimensions, however, the dimensions are not equally covered and efforts should be made to balance all three dimensions [37].

### Observation 3: Economic sustainability topics are of increasing importance in organizations

The mean probability of most economic sustainability topics is increasing, indicating that–while economic, social, and environmental sustainability overall are covered equally in sustainability reports–mentions of economic sustainability are increasing. This finding confirms previous results concerning an increasing relevance of economic topics in sustainability reports [2] that might be a consequence of the 2008 financial crisis [1]. The mean probability of all environmental sustainability topics and the mean probability of all social sustainability topics have been largely stable since 1999; however, these two areas’ mean probabilities in all of the reports in our analysis are higher than that of all economic sustainability topics. One reason for this result might be seen in economic pressures like that seen in the Europe crises [38,39], the concerns about the Chinese economy [40], or organizations’ growing interest in digital innovation and transformation in their businesses [41]. In this regard, the data might confirm that organizations prioritize economic concerns during crises and that ecological and social interests are more likely to be considered in stable economic times [42,43]. However, research indicates that sustainability transformation can also offer economic potential for organizations and that digital technologies in particular can open up new business opportunities and business models in areas of environmental and social sustainability [37,44,45] like smart houses and energy supply solutions [46]. Consequently, organizations should leverage the economic potential of including environmental and social sustainability in their activities.

### Observation 4: As to environmental sustainability, organizations report on emissions and energy consumption

Diverse measures for environmental sustainability have been discussed in the literature, including air emissions, energy use, resource depletion, waste, and water use [14]. Our study reveals that organizations predominantly report on their emissions and energy consumption data. Emissions and consumption are also the most frequently mentioned environmental issues found in a previous analysis, but in that analysis consumption data for energy and water were reported equally often [1]. In our results, energy was more probable than water in the context of environmental sustainability performance. We found a specific topic on water, but this topic focused on waste water. Consequently, we conclude that organizations should increase their range of measures for environmental sustainability by, for instance, additionally reporting on fuel and paper consumption, waste, and emission of certain gases [1].

### Observation 5: Biodiversity and renewable energy sources receive little attention in reports by organizations

Research has identified both biodiversity and renewable energy sources as important aspects of environmental sustainability [14,47], but these topics were absent or rare in the sustainability reports we analysed. The probability of the term *biodiversity* is close to zero, and we found no evidence that the term occurs in the context of general environmental sustainability reporting. One reason for this rarity might be the complexity of biodiversity, as the related impacts of some of organizations’ actions are often distant in time and space [48]. Furthermore, other than some well-known threats to biodiversity, such as pollution, many threats are not yet fully understood [48], making it difficult for organization to address the issue. Another explanation might be that loss of biodiversity is a result of environmentally unsustainable behaviour, including over-abstraction of water, increasing demand for resources, and rising consumption levels [48], topics that are well-covered in the sustainability reports. Terms that relate to renewable energy are also rare in the reports. Our study suggests that organizations have not taken significant action to invest in renewable energy, nor are they reporting on projects to come. Investigations on why this is the case in terms of whether organizations see sufficient potential in renewable energy and biodiversity and plan to adopt it in the future would be useful.

### Observation 6: Regarding social sustainability, organizations report on labour practices

The literature has discussed diverse issues concerning social sustainability, such as employee training programs, health and prevention programs, stakeholder involvement, customer satisfaction, and sponsorship [13,14]. Our study also supports the importance of topics like safety, work time, diversity, and development. These topics tend to cover the indicators mentioned in the global reporting principles and standards under the sub-category of labour practices and decent work, and organizations seem to cover the most important human rights in their reports. Compared to all 42 topics, which we analysed, two of the topics related to employees are among the five most probable topics (having the highest mean probability of occurring in a sustainability report), so employees play an important role in organizational sustainability reports. However, organizations should scope their sustainability initiatives beyond legal requirements [37] in order to differentiate themselves from their competitors. In particular, continuing with efforts concerning labour practices beyond existing regulations can greatly improve an employer’s profile and attract top talent on the competitive global job market [49].

### Observation 7: Customer orientation is in organizations’ focus

The literature has identified several motives for organizations to engage in sustainability transformations, including regulations [14,20,21], pressure from customers [21], and new market creation [45]. Against this background, our analysis revealed a dominant topic related to customer-orientation. The probable terms of *customer*, *responsibility*, *satisfaction*, *information*, *online*, and *survey* show that organizations are concerned with customer satisfaction and use online surveys to measure it. These findings suggest that customer orientation is a valid strategy for organizations, as they report on their sustainability initiatives with respect to their customers’ perspective. Drawing from the data, we conclude that organizations should scope sustainability initiatives in order to consider a wider range of stakeholders. Stakeholder theory, in particular, shows that multiple views should be balanced in order to achieve business success over the mid- to long-term [50]. Sustainability research has also shown that corporate sustainability requires that one considers interactions with and value creation for all stakeholders [37,51].

### Observation 8: Sponsorship activities for social sustainability focus on schools and education

Sponsorship activities are part of organizations’ social sustainability practices [14]. Since our analysis reveals a focus of such sponsorship on funds for schools and education, we find that decision makers appear to believe in the role of education in improving (social) sustainability over the long term [52]. Building on our data, we conclude that, in addition to their current activities, organizations should invest in their employees’ education as well in order to achieve bottom-up support of their sustainability activities, which has been shown to help organizations adopt sustainable practices [23].

### Observation 9: Economic sustainability reporting is based on financial data

The topics on economic sustainability focus on financial data particularly that which is part of the corporate balance sheet. Our study did not find topics on practices like compliance or on codes of conduct, both of which have been identified as valuable in the effort to sustain economic success. We argue that sustainability reports should be enriched by statements on how to sustain and develop economic results in pursuit of economic sustainability [37].

### Observation 10: Sustainability actions are both general and context-specific in nature

Research on sustainability transformations has identified a number of action potentials, such as guiding behaviour by sense-making and sustainable practices [53]. Building on the data from the sustainability reports of 3,900 companies, our study reveals a broad range of topics covered in sustainability reports, some of which are related to certain industries or certain geographic regions, while others are well distributed among industries and regions. In this regard, our study confirms previous assumptions about industry-specific practices [3]. We further assume that the differences in industries and regions might be due to different stakeholders, e.g. different national initiatives. Also previous research showed that the content of sustainability reports is influence by the organization’s stakeholders [1]. Therefore, we argue that future research and practice should be more specific in characterizing and understanding the context of sustainability behaviour.

In several cases, we can make assumptions concerning why a certain industry or region focuses on a particular topic. For instance, *green production* is most likely to be a topic in reports from companies in Asia, especially those in the technology hardware, consumer durables, and equipment industries, with which companies in China, Japan, and Taiwan are typically associated. Therefore, we see a link among the continent, the industry, and the most probable terms in the topic. The *production and packaging* topic is most likely to occur in reports from companies in Europe, possibly because of the European directive on packaging and packaging waste that aims to improve packaging’s environmental performance. Another example is *sustainability projects*, which are most likely to occur in German reports, possibly because of the German Energy Transition (“Energiewende”), a movement toward alternative energy sources that started in the 1970s and gained popularity in 2011 [54]. Therefore, we agree with the statement from Liew et al. [3] that sustainability practices are industry-specific. We also show how different stakeholders—particularly governments through regulations—influence the content of sustainability reports [1].

We summarize these ten observations and ten recommendations in Table 6.

**Table 6:** Summary of observations and recommendations.

#	Observation	Propositions
1	Organizations report on environmental, social, and economic sustainability.	Organizations should distinguish among the three dimensions of sustainability on the operational level but focus on integrating the three dimensions on a strategic level.
2	Topics on environmental, social, and economic sustainability are equally distributed.	Organizations should balance social, environmental, and economic dimensions of sustainability in their choice of sustainability-related activities.
3	Economic sustainability topics are of increasing importance for organizations.	Organizations should leverage the economic potential of including environmental and social sustainability in their activities.
4	As to environmental sustainability, organizations report on emissions and energy consumption.	Organizations should increase their range of measures of environmental sustainability by, for instance, additionally reporting on fuel and paper consumption, waste, and emission of certain gases.
5	Biodiversity and renewable energy sources receive little attention in reports by organizations.	Researchers should investigate whether organizations see sufficient potential in renewable energy and biodiversity and plan to adopt it in the future.
6	Regarding social sustainability, organizations report on labour practices.	Organizations should continue their efforts concerning labour practices beyond existing regulations in order to improve their profiles as employers.
7	Customer orientation is in organizations’ focus.	Organizations should consider their interaction with and value creation for all stakeholders.
8	Sponsorship activities for social sustainability focus on schools and education.	Organizations should invest in their employees’ sustainability-related education.
9	Economic sustainability reporting is based on financial data.	Organizations should enrich their reports with statements on how to sustain and improve economic results.
10	Sustainability actions are both general and context-specific in nature.	Researchers should be more specific in characterizing and explaining the context of sustainability behaviour.

## Conclusion

Increasing numbers of organizations are publishing sustainability reports about their sustainability practices [2]. The present work used topic-modelling techniques to analyse 9,514 sustainability reports published by organizations between 1999 and 2015 and derives ten specific propositions to guide future research and practice.

More specifically, we identified forty-two topics related to sustainability that are distributed approximately equally in the areas of environmental, social, economic, and general sustainability. We showed that topics related to environmental sustainability consist mainly of emissions and consumption, particularly related to energy; biodiversity and renewable energy do not appear in our results. The focus of social sustainability is on employees, but customer orientation and sponsorship are also covered. In addressing economic sustainability, organizations tend simply to present their financial data. We also show the influence of industry and country on the content of the topics.

We advise organizations to balance their activities in and to use the potential of all three dimensions of sustainability, to increase their measures for environmental sustainability, to continue with their efforts concerning labour practices, to consider their interactions with all stakeholders, to invest in their employees’ education concerning sustainability, and to provide information in their sustainability reports on how to sustain and develop their economic results. Researchers are advised to investigate why organizations have not focused on biodiversity or renewable energy and to be precise on the contexts of the sustainability behaviours they examine.

Our work is not without limitations. We used text-mining techniques that reduce the content of the documents to simple collections of terms, so our findings depend on our interpretation of the results. Particularly, the labelling of the topics is based on the subjective opinion of the authors and other researchers might come up with different labels. Still, we are convinced that our labels represent the content of each topic (based on the most probable terms that describe this topic) well and thus, also other labels would not have a big influence on our findings and observations. Nevertheless, we encourage other researchers to evaluate our results through an in-depth qualitative analysis of sustainability reports. In addition, our data sources are sustainability reports that organizations publish to report on their sustainability activities, but they are also used as marketing instruments [1], so they might not reflect corporate sustainability practices in all details. Furthermore, we received these reports from one single source, the GRI database, thus, there might exist a certain bias in the data. Future research can address this limitation by complementing our findings with interviews of those who participate in sustainability activities in organizations or use another data source such as the Corporate Register, a database with more than eighty thousand corporate responsibility reports (http://corporateregister.com). To our best knowledge, our application of LDA in the context of sustainability reports is new, so questions could arise regarding its reliability. While the use of LDA is not without risk, we are confident that, in this case, it provided valuable insights.

Despite its risks, we propose that other researchers use LDA in their research on sustainability reports or organizational reports in general, as its use in these contexts has several advantages compared to manual coding techniques: First, LDA allows the researcher to take a large amount of data into consideration and opposed to manual coding, the data analysis costs are minimal [32]; in our case, it allowed us to provide a broad picture of sustainability practices over several industries and years. Second, LDA requires no restrictions on the content of the topics, such as the requirement that one focuses on certain indicators as previous studies [8] have done; we had only to restrict the number of topics to be modelled. Third, LDA allows the resulting topics to be used for further analysis, such as we did in analysing the topic distribution over industries, years, and regions, restricted only to the nearly forty industries that published their sustainability reports on the GRI database. For example, the number of reports from the toy industry might otherwise have kept it from being the focus of any sustainability research, but using LDA allowed it to be included, and our analysis revealed two topics, *work time* and *corporate responsibility activities*, that were highly probable in reports from this industry. Fourth, applying LDA provides a broad overview of the many definitions and conceptualizations of sustainability that exist in organizations.

Our research can also guide practitioners in their sustainability activities, as it provides a comprehensive overview of potential sustainability-related efforts among the ten recommendations.

The study contributes to research in two ways. First, researchers can use our results to either explore the topics that resulted from the analysis or to explore the reasons for missing topics. Second, we propose a new technique for analysing sustainability reports or corporate reports in general that other researchers might use to analyse other types of documents.

## Appendix

Table 7 provides on overview about all topics, including the label (if related to sustainability), the most probable terms and the sustainability dimension.

**Table 7:** Overview on all 70 topics including the twenty most occurring ordered by their probability of occurrence.

Topic No.	Label	Most probable terms	Environmental Sustainability	Social Sustainability	Economic Sustainability	General Sustainability	Not related to sustainability / Not relevant for report
0	**Employee safety**	employee 3.56%; safety 3.53%; management 2.13%; health 1.87%; training 1.32%; performance 1.24%; risk 1.14%; environmental 1.01%; corporate 0.97%; environment 0.92%; community 0.90%; system 0.88%; operation 0.87%; process 0.86%; business 0.78%; local 0.70%; compliance 0.70%; supplier 0.66%; standard 0.65%; ensure 0.63%		x			
1	**Organizational development program**	program 3.33%; organization 2.30%; development 1.95%; management 1.49%; social 1.46%; employee 1.07%; center 1.05%; labor 0.91%; responsibility 0.90%; system 0.84%; service 0.80%; training 0.78%; quality 0.73%; department 0.64%; public 0.61%; project 0.56%; resource 0.55%; information 0.51%; order 0.49%; activity 0.49%		x			
2		gas 4.36%; oil 3.75%; production 1.59%; energy 1.37%; project 1.28%; natural 1.02%; development 0.97%; operation 0.84%; million 0.79%; total 0.78%; refinery 0.77%; pipeline 0.76%; emission 0.71%; exploration 0.67%; petroleum 0.64%; safety 0.57%; field 0.55%; fuel 0.55%; activity 0.54%; hse 0.53%					x
3	**Nuclear power**	development 1.10%; nuclear 0.92%; system 0.86%; management 0.86%; activity 0.83%; social 0.69%; production 0.65%; state 0.61%; project 0.60%; corporate 0.57%; region 0.56%; employee 0.54%; safety 0.53%; information 0.53%; work 0.49%; environmental 0.48%; power 0.46%; unit 0.45%; reporting 0.43%; corporation 0.43%				x	
4	**Employee diversity**	program 2.11%; employee 2.05%; corporate 1.60%; community 1.43%; business 1.36%; responsibility 1.20%; global 1.02%; organization 0.92%; support 0.69%; diversity 0.68%; work 0.65%; environmental 0.65%; commitment 0.54%; social 0.52%; service 0.50%; citizenship 0.47%; effort 0.46%; team 0.46%; leadership 0.46%; initiative 0.46%		x			
5	**Work time**	people 1.59%; work 1.27%; employee 1.21%; time 0.90%; team 0.90%; make 0.78%; day 0.74%; life 0.56%; event 0.53%; child 0.51%; part 0.50%; family 0.45%; working 0.45%; good 0.42%; office 0.42%; member 0.42%; staff 0.40%; manager 0.39%; care 0.38%; environment 0.38%		x			
6		port 1.92%; marine 1.83%; ship 1.67%; vessel 1.52%; logistics 1.32%; service 1.21%; shipping 0.99%; offshore 0.99%; group 0.97%;; terminal 0.92%; container 0.83%; freight 0.79%; fuel 0.78%; system 0.77%; industry 0.72%; fleet 0.66%; sea 0.65%; cargo 0.63%; oil 0.62%; management 0.59%					x
7		steel 4.27%; product 1.81%; metal 1.74%; production 1.53%; material 1.32%; aluminium 1.28%; plant 1.24%; iron 0.98%; emission 0.92%; process 0.90%; energy 0.69%; industry 0.65%; raw 0.62%; work 0.60%; furnace 0.58%; customer 0.58%; arcelormittal 0.55%; recycling 0.52%; hydro 0.50%; operation 0.49%					x
8	**Stakeholder issues**	stakeholder 1.23%; issue 1.20%; business 1.13%; policy 0.99%; human 0.85%; social 0.85%; country 0.85%; principle 0.85%; global 0.83%; information 0.81%; responsibility 0.77%; standard 0.72%; work 0.67%; corporate 0.67%; reporting 0.64%; process 0.62%; product 0.61%; tobacco 0.57%; international 0.55%; impact 0.51%				x	
9	**Financial data 1**	eur 2.76%; group 1.74%; euro 1.62%; thousand 1.37%; board 1.20%; share 1.04%; financial 0.80%; market 0.79%; asset 0.75%; supervisory 0.72%; member 0.71%; general 0.68%; consolidated 0.59%; cost 0.58%; activity 0.56%; amount 0.56%; shareholder 0.53%; tax 0.52%; dec 0.51%; annual 0.51%			x		
10	**Green supplier**	supplier 1.81%; product 1.45%; employee 1.19%; technology 1.12%; global 1.05%; chain 1.00%; supply 0.98%; energy 0.91%; data 0.89%; program 0.74%; emission 0.64%; solution 0.60%; information 0.54%; business 0.51%; system 0.46%; content 0.45%; management 0.42%; material 0.42%; world 0.40%; human 0.40%	x				
11	**Social sustainability data**	social 0.82%; program 0.82%; sustainability 0.77%; management 0.74%; total 0.71%; employee 0.70%; area 0.65%; project 0.62%; environmental 0.58%; unit 0.53%; process 0.50%; action 0.46%; gri 0.46%; state 0.46%; development 0.40%; indicator 0.38%; result 0.37%; service 0.35%; activity 0.34%; investment 0.33%		x			
12	**Investment**	share 3.65%; limited 2.22%; group 2.11%; investment 1.22%; director 1.03%; interest 0.98%; annual 0.95%; million 0.91%; asset 0.89%; subsidiary 0.77%; statement 0.77%; continued 0.75%; dividend 0.69%; option 0.68%; rate 0.67%; date 0.65%; ordinary 0.64%; holding 0.64%; note 0.61%; cash 0.59%			x		
13		energy 5.29%; power 4.67%; electricity 2.34%; plant 2.30%; generation 1.37%; gas 1.32%; wind 1.10%; mw 0.93%; emission 0.91%; project 0.90%; electric 0.84%; renewable 0.79%; supply 0.72%; capacity 0.66%; system 0.65%; transmission 0.63%; grid 0.63%; million 0.63%; station 0.60%; customer 0.60%					x
14	**Organizational sustainability**	business 0.86%; employee 0.72%; management 0.71%; sustainability 0.62%; limited 0.58%; plc 0.56%; initiative 0.54%; unit 0.52%; st 0.50%; corporate 0.43%; award 0.43%; fully 0.42%; policy 0.41%; performance 0.40%; development 0.39%; growth 0.37%; process 0.35%; environment 0.34%; community 0.34%; stakeholder 0.34%				x	
15		mining 2.71%; mine 2.12%; resource 1.72%; mineral 1.50%; us$ 1.43%; production 1.14%; ore 1.10%; project 1.02%; million 0.99%; gold 0.93%; exploration 0.86%; reserve 0.82%; cost 0.78%; diamond 0.77%; coal 0.75%; price 0.74%; operation 0.73%; share 0.64%; tonne 0.57%; total 0.49%					x
16	**Environmental sustainability performance**	percent 2.17%; program 1.92%; energy 1.59%; facility 1.07%; goal 0.77%; community 0.72%; state 0.69%; emission 0.68%; environmental 0.66%; organization 0.53%; effort 0.52%; employee 0.50%; water 0.49%; gas 0.47%; center 0.44%; ton 0.44%; system 0.43%; reduce 0.40%; metric 0.40%	x				
17		patient 1.91%; healthcare 1.61%; health 1.56%; product 1.49%; pharmaceutical 1.18%; medicine 1.09%; care 0.95%; research 0.90%; medical 0.90%; disease 0.81%; hospital 0.77%; treatment 0.76%; clinical 0.70%; access 0.65%; country 0.64%; drug 0.62%; site 0.50%; vaccine 0.49%; million 0.45%					x
18	**Financial data 2**	share 2.20%; group 1.55%; financial 1.35%; director 1.24%; board 1.18%; information 0.84%; statement 0.82%; document 0.81%; meeting 0.73%; million 0.73%; consolidated 0.73%; shareholder 0.69%; capital 0.67%; registration 0.61%; asset 0.61%; total 0.60%;option 0.60%; euro 0.58%; plan 0.55%; amount 0.52%			x		
19	**Management**	management 2.66%; system 1.09%; business 1.01%; employee 0.94%; activity 0.76%; sustainability 0.63%; social 0.62%; program 0.56%; green 0.55%; global 0.54%; krw 0.52%; support 0.52%; growth 0.52%; issue 0.49%; performance 0.49%; customer 0.44%; overseas 0.43%; stakeholder 0.41%; information 0.40%; partner 0.39%		x			
20	**Customer orientation**	customer 12.24%; service 4.68%; employee 2.79%; business 2.54%; corporate 2.07%; product 1.59%; responsibility 1.48%; satisfaction 0.85%; information 0.68%; online 0.64%; stakeholder 0.63%; survey 0.62%; million 0.56%; number 0.55%; group 0.54%; network 0.50%; offer 0.48%; solution 0.42%; contact 0.42%; post 0.42%		x			
21	**Production**	product 2.44%; plant 2.23%; chemical 2.15%; cement 1.98%; material 1.87%; production 1.33%; safety 1.30%; emission 1.26%; site 1.08%; industry 0.87%; raw 0.80%; business 0.74%; process 0.70%; million 0.68%;; el 0.66%; customer 0.65%; concrete 0.60%;industrial 0.54%; dow 0.51%; ton 0.48%	x				
22		pg 1.90%; employee 1.33%; fiscal 1.25%; product 0.96%; food 0.90%; health 0.85%; program 0.82%; water 0.80%; global 0.77%; safety 0.60%; system 0.52%; process 0.49%; energy 0.49%; child 0.46%; facility 0.45%; management 0.44%; consumer 0.41%; corporate 0.39%; social 0.39%; project 0.38%					x
23		property 5.82%; investment 2.12%; portfolio 1.45%; fund 1.29%; tenant 1.29%; unit 1.21%; asset 1.12%; centre 1.10%; shopping 1.06%; retail 1.01%; management 0.93%; rental 0.92%; interest 0.87%; income 0.87%; office 0.81%; rate 0.80%; total 0.78%; million 0.76%; linked 0.71%; estate 0.67%					x
24		corporation 7.96%; philippine 1.51%; business 1.08%; globe 1.04%; land 0.94%; development 0.89%; bell 0.72%; sustainability 0.68%; te 0.66%; president 0.66%; corporate 0.65%; management 0.65%; city 0.55%; million 0.50%; fax 0.46%; performance 0.44%; integrated 0.43%; total 0.43%; cebu 0.40%; director 0.40%					x
25	**Sustainable development**	group 6.56%; employee 1.14%; development 1.02%; sustainable 0.91%; social 0.89%; country 0.81%; environmental 0.74%; indicator 0.66%; site 0.64%; local 0.64%; csr 0.59%; commitment 0.58%; action 0.54%; energy 0.51%; policy 0.50%; initiative 0.47%; training 0.45%; project 0.44%; activity 0.42%; performance 0.40%		x			
26	**Development program**	programme 4.65%; organisation 3.07%; development 1.87%; centre 1.47%; group 1.41%; social 1.38%; staff 1.34%; management 1.17%; service 0.95%; labour 0.92%; training 0.89%; system 0.88%; department 0.69%; sustainable 0.68%; environmental 0.68%; quality 0.65%; responsibility 0.61%; aim 0.57%; work 0.56%; policy 0.45%$		x			
27		vehicle 3.78%; car 1.43%; fuel 1.32%; engine 1.25%; emission 1.12%; plant 1.11%; safety 0.96%; technology 0.88%; system 0.85%; automotive 0.82%; supplier 0.80%; motor 0.78%; product 0.78%; production 0.74%; customer 0.68%; truck 0.60%; part 0.60%; renault 0.58%; sale 0.55%; material 0.54%					x
28		community 1.85%; operation 1.75%; mine 1.66%; mining 1.49%; site 0.93%; project 0.89%; development 0.84%; water 0.83%; local 0.82%; coal 0.74%; copper 0.64%; management 0.64%; area 0.63%; health 0.55%; environmental 0.54%; land 0.52%; safety 0.50%; business 0.47%; employee 0.46%; emission 0.43%					x
29	**Financial data 3**	group 1.73%; share 1.45%; board 1.33%; annual 1.07%;; eur 1.03%; total 1.02%; financial 0.91%; sek 0.79%; market 0.76%; risk 0.68%; million 0.63%; member 0.61%; sale 0.58%; operation 0.58%; pension 0.57%; operating 0.56%; change 0.54%; nok 0.53%; liability 0.51%; tax 0.50%			x		
30	**Sustainability activities**	group 1.35%; activity 0.78%; system 0.69%; area 0.65%; total 0.63%; management 0.61%; sustainability 0.57%; service 0.54%; euro 0.52%; project 0.51%; initiative 0.42%; environmental 0.41%; specific 0.39%; training 0.37%; work 0.37%; energy 0.36%; social 0.35%; relation 0.35%; level 0.34%; process 0.34%				x	
31	**Sustainability program**	program 2.21%; unit 0.99%; sustainability 0.93%; total 0.74%; area 0.74%; employee 0.74%; business 0.67%; management 0.65%; activity 0.61%; air 0.59%; community 0.57%; development 0.56%; commissioner 0.54%; corporate 0.51%; sdn 0.50%; pln 0.48%; performance 0.48%; csr 0.48%; environment 0.45%; director 0.43%				x	
32	**Environmental sustainability data**	emission 2.38%; sustainability 2.08%; reporting 1.90%; performance 1.82%; data 1.72%; gri 1.64%; total 1.56%; energy 1.40%; environmental 1.17%; indicator 1.12%; waste 1.04%; employee 1.04%; consumption 1.03%; scope 0.96%; management 0.90%; stakeholder 0.89%; material 0.84%; assurance 0.83%; impact 0.76%; number 0.72%	x				
33	**CSR activity**	group 2.43%; csr 2.00%; activity 1.41%; business 1.24%; management 1.09%; environmental 0.97%; system 0.93%; employee 0.91%; fiscal 0.84%; information 0.73%; corporate 0.66%; society 0.57%; environment 0.49%; social 0.45%; initiative 0.44%; effort 0.44%; global 0.43%; overseas 0.42%; measure 0.42%; compliance 0.41%				x	
34	**Business development**	business 2.99%; market 1.93%; growth 1.59%; service 1.44%; client 1.21%; solution 1.14%; global 1.03%; management 0.88%; technology 0.76%; industry 0.71%; investment 0.69%; development 0.69%; strategy 0.68%; country 0.66%; performance 0.61%; sector 0.61%; key 0.58%; revenue 0.53%; world 0.50%; strategic 0.46%			x		
35	**Production & Packaging**	product 4.12%; packaging 2.19%; consumer 1.97%; water 1.61%; material 1.26%; business 1.06%; brand 1.04%; waste 1.03%; supplier 0.88%; beverage 0.81%; manufacturing 0.75%; plant 0.67%; safety 0.62%; recycling 0.58%; health 0.57%; production 0.56%; raw 0.56%; life 0.51%; plastic 0.51%; chain 0.48%	x				
36		food 4.43%; product 1.91%; production 1.24%; farmer 1.22%; farm 0.92%; agricultural 0.81%; feed 0.78%; crop 0.72%; animal 0.71%; nutrition 0.70%;; quality 0.68%; agriculture 0.67%; sugar 0.66%; oil 0.66%; palm 0.63%; farming 0.60%;fish 0.58%; coffee 0.56%;dairy 0.55%; plant 0.53%					x
37		hotel 5.34%; guest 1.49%; resort 1.46%; gaming 1.36%; tourism 1.25%; crown 1.15%; restaurant 0.95%; blue 0.87%; casino 0.85%; room 0.76%; travel 0.73%; label 0.73%; property 0.66%; international 0.65%; game 0.64%; brand 0.63%; leisure 0.63%; responsible 0.63%; lub 0.62%; hospitality 0.62%					x
38	**Annual meeting**	share 1.91%;director 1.83%; annual 1.50%; group 1.36%; shareholder 1.13%; meeting 1.06%; resolution 0.95%; committee 0.92%; board 0.88%; limited 0.87%; financial 0.86%; general 0.84%; ordinary 0.82%; proxy 0.81%; number 0.76%; act 0.61%; statement 0.58%; audit 0.57%; term 0.53%; integrated 0.52%				x	
39	**Financial data 4**	financial 3.25%; asset 2.52%; statement 1.62%; group 1.37%; liability 1.36%; cost 1.19%; amount 1.16%; loss 1.15%; cash 1.14%; fair 1.11%; income 1.07%; tax 1.03%; note 0.97%; risk 0.95%; interest 0.94%; recognised 0.90%; rate 0.82%; profit 0.75%; impairment 0.68%; annual 0.64%			x		
40		airport 5.44%; air 2.87%; airline 2.43%; aircraft 2.42%; passenger 1.76%; flight 1.32%; aviation 1.31%; service 1.26%; fuel 1.26%; international 0.91%; operation 0.78%; emission 0.73%; noise 0.71%; safety 0.67%; cargo 0.63%; ground 0.60%; travel 0.54%; environmental 0.50%; number 0.49%; transport 0.48%					x
41	**General sustainability**	sustainability 5.53%; sustainable 1.74%; business 1.50%; energy 1.14%;; impact 0.95%; stakeholder 0.94%; performance 0.86%; strategy 0.82%; environmental 0.82%; community 0.82%; change 0.79%; water 0.79%; development 0.78%; approach 0.76%; carbon 0.75%; climate 0.73%; issue 0.70%; people 0.70%; target 0.66%; engagement 0.62%				x	
42		group 2.0%; share 1.7%; director 1.4%; performance 1.3%; million 1.0%; executive 0.9%; remuneration 0.9%; limited 0.9%; committee 0.7%; board 0.7%; plan 0.6%; tax 0.6%; financial 0.6%; business 0.6%; cash 0.6%; annual 0.5%; award 0.5%; net 0.5%; option 0.5%; total 0.5%					x
43	**Employee responsibility**	employee 1.78%; group 1.67%; corporate 0.86%; management 0.84%; activity 0.76%; responsibility 0.72%; project 0.64%; system 0.64%; social 0.61%; training 0.56%; work 0.45%; environmental 0.43%; principle 0.43%; product 0.43%; total 0.43%; environment 0.42%; support 0.41%; business 0.40%; member 0.37%; information 0.37%		x			
44	**Corporate social responsibility**	corporate 1.25%; management 1.00%; social 0.95%; activity 0.87%; responsibility 0.76%; information 0.72%; group 0.69%; area 0.66%; environmental 0.65%; commitment 0.59%; project 0.57%; service 0.55%; action 0.53%; system 0.52%; plan 0.50%; total 0.49%; work 0.48%; employee 0.48%; supplier 0.45%; process 0.39%		x			
45	**Green production**	product 4.34%; environmental 2.83%; production 1.45%; system 1.26%; material 1.25%; energy 1.24%; manufacturing 1.11%; waste 0.99%; technology 0.93%; customer 0.85%; site 0.80%; reduction 0.73%; plant 0.70%; recycling 0.69%; emission 0.68%; substance 0.63%; process 0.63%; supplier 0.62%; sale 0.62%; impact 0.62%	x				
46	**Management summary**	pro 3.17%; management 1.28%; profi 0.95%; board 0.84%; business 0.83%; fl 0.80%; risk 0.77%; benefi 0.73%; signifi 0.70%; director 0.63%; scal 0.55%; effi 0.55%; market 0.53%; gures 0.52%; reporting 0.51%; governance 0.51%; net 0.48%; information 0.46%; cantly 0.45%; product 0.44%			x		
47		road 2.80%; transport 2.15%; rail 1.71%; service 1.64%; safety 1.17%; public 1.11%; work 1.08%; train 1.08%; infrastructure 1.04%; station 1.02%; network 0.98%; project 0.92%; traffic 0.90%; concession 0.84%; railway 0.84%; system 0.80%; transportation 0.73%; line 0.71%; bus 0.69%; passenger 0.69%					x
48	**Sustainability projects**	group 1.69%; employee 1.53%; management 1.16%; sustainability 1.05%; energy 0.75%; corporate 0.64%; measure 0.57%; responsibility 0.57%; project 0.53%; protection 0.48%; business 0.47%; process 0.47%; system 0.47%; work 0.47%; area 0.44%; environmental 0.42%; board 0.41%; percent 0.41%; important 0.40%; consumption 0.38%				x	
49		risk 2.85%; asset 1.47%; credit 1.43%; loan 1.25%; capital 1.23%; bank 1.19%; financial 1.05%; group 1.03%; management 0.96%; income 0.95%; interest 0.89%; security 0.88%; market 0.86%; investment 0.84%; total 0.83%; loss 0.80%; fair 0.70%; million 0.67%; portfolio 0.67%; rate 0.66%					x
50	**Corporate responsibility**	group 1.74%; business 1.53%; community 1.24%; cr 1.22%; cooperative 0.99%; people 0.95%; target 0.76%; programme 0.76%; support 0.63%; work 0.59%; data 0.58%; responsibility 0.56%; carbon 0.54%; employee 0.49%; waste 0.49%; plc 0.49%; performance 0.49%; tonne 0.46%; change 0.45%; energy 0.44%				x	
51		insurance 4.73%; investment 3.12%; risk 2.32%; life 2.00%; fund 1.78%; financial 1.75%; group 1.44%; asset 1.30%; management 1.26%; business 1.14%; claim 1.13%; premium 0.98%; product 0.87%; policy 0.80%; equity 0.70%; market 0.68%; change 0.67%; contract 0.61%; capital 0.57%; portfolio 0.54%					x
52		bank 8.17%; financial 2.46%; banking 2.10%; branch 1.42%; loan 1.39%; credit 1.28%; social 1.03%; risk 0.98%; investment 0.98%; client 0.92%; finance 0.89%; service 0.88%; employee 0.84%; product 0.83%; fund 0.82%; environmental 0.75%; business 0.70%; management 0.63%; project 0.62%; policy 0.61%					x
53	**Building construction**	construction 4.09%; building 4.00%; project 3.99%; energy 1.66%; development 1.57%; green 1.41%; design 1.24%; site 1.21%; office 0.92%; work 0.86%; housing 0.81%; land 0.79%; home 0.75%; engineering 0.73%; sustainable 0.71%; environmental 0.70%; award 0.70%; water 0.66%; area 0.66%; material 0.57%	x				
54		million 2.27%; share 1.33%; financial 1.32%; income 1.32%; net 1.30%; asset 1.29%; sale 1.02%; tax 1.00%; cash 0.94%; statement 0.92%; consolidated 0.90%; cost 0.85%; total 0.82%; note 0.70%; rate 0.69%; business 0.68%; loss 0.66%; plan 0.66%; market 0.66%					x
55		university 2.82%; research 2.16%; student 1.88%; member 1.38%; science 1.15%; staff 1.13%; education 1.01%; campus 1.01%; engineering 0.87%; national 0.69%; international 0.65%; engineer 0.59%; service 0.59%; conference 0.59%; commission 0.58%; court 0.52%; academic 0.52%; division 0.51%; judicial 0.51%; public 0.50%					x
56	**Development**	development 1.70%; employee 1.04%; management 0.77%; operation 0.73%; programme 0.67%; platinum 0.62%; project 0.56%; risk 0.52%; water 0.47%; level 0.45%; total 0.45%; million 0.45%; sustainable 0.44%; community 0.43%; issue 0.40%; performance 0.40%; capital 0.39%; area 0.38%; process 0.38%; skill 0.37%				x	
57		medium 2.94%; beer 1.65%; alcohol 1.42%; brewery 1.36%; content 0.95%; responsible 0.94%; production 0.90%; advertising 0.83%; digital 0.77%; drinking 0.70%; brand 0.68%; marketing 0.64%; wine 0.63%; heineken 0.63%; television 0.61%; print 0.57%; music 0.55%; tv 0.53%; million 0.52%; consumption 0.52%					x
58	**Financial data 5**	board 2.36%; million 1.54%; supervisory 1.48%; delta 1.10%; annual 1.08%; share 1.04%; nv 1.03%; result 0.99%; member 0.81%; van 0.78%; management 0.72%; lloyd 0.70%; statement 0.70%; total 0.69%; risk 0.67%; policy 0.62%; dsm 0.55%; financial 0.55%; remuneration 0.55%; die 0.55%			x		
59		pro 1.55%; work 0.81%; man 0.73%; area 0.43%; develop 0.41%; employ 0.33%; board 0.33%; number 0.32%; member 0.30%; social 0.30%; part 0.29%; total 0.29%; level 0.28%; ability 0.27%; holder 0.27%; result 0.26%; economic 0.26%; sustain 0.26%; port 0.25%; ship 0.25%					x
60	**School sponsorship**	community 2.78%; school 2.45%; project 2.28%; education 1.93%; child 1.70%; development 1.68%; support 1.66%; foundation 1.28%; health 1.16%; initiative 1.11%; student 1.04%; world 0.86%; international 0.84%; family 0.83%; society 0.81%; local 0.81%; people 0.78%; partnership 0.76%; country 0.70%; environment 0.69%		x			
61		store 4.61%; product 2.39%; supplier 1.71%; factory 1.62%; brand 1.36%; retail 1.29%; sale 1.06%; chain 0.93%; audit 0.79%; associate 0.70%; supply 0.68%; worker 0.64%; good 0.56%; retailer 0.55%; production 0.52%; customer 0.51%; country 0.50%; working 0.50%; total 0.50%; textile 0.49%					x
62		city 2.93%; council 2.29%; community 1.16%; service 0.99%; centre 0.80%;; annual 0.80%; plan 0.72%; gold 0.72%; park 0.70%; public 0.67%; coast 0.67%; local 0.67%; government 0.63%; committee 0.60%; aboriginal 0.52%; olympic 0.50%; asset 0.46%; event 0.46%; development 0.45%; activity 0.45%					x
63	**Water management**	water 9.53%; waste 3.26%; plant 2.18%; environmental 1.48%; treatment 1.24%; management 0.95%; system 0.93%; service 0.85%; area 0.83%; environment 0.81%; quality 0.80%; wastewater 0.80%; total 0.76%; supply 0.72%; energy 0.64%; project 0.57%; local 0.49%; work 0.49%; resource 0.48%; emission 0.43%	x				
64		service 3.72%; mobile 2.63%; network 2.40%; communication 1.28%; customer 1.26%; telecommunication 1.15%; data 1.15%; internet 1.08%; telekom 0.96%; access 0.87%; technology 0.85%; information 0.78%; phone 0.74%; million 0.64%; broadband 0.57%; digital 0.54%; ict 0.53%; operator 0.53%; employee 0.49%; call 0.49%					x
65		forest 3.84%; paper 3.32%; mill 1.85%; product 1.84%; wood 1.55%; ulp 1.16%; forestry 1.08%; environmental 0.83%; production 0.81%; area 0.77%; management 0.69%; sustainable 0.63%; plantation 0.63%; certification 0.57%; land 0.55%; material 0.52%; million 0.51%; tree 0.51%; industry 0.51%; timber 0.50%					x
66	**Financial data 6**	group 3.17%; integrated 1.25%; committee 1.17%; risk 0.97%; financial 0.92%; management 0.85%; business 0.83%; board 0.82%; performance 0.71%; executive 0.69%; annual 0.65%; employee 0.63%; director 0.55%; share 0.53%; remuneration 0.53%; review 0.51%; million 0.46%; continued 0.46%; operation 0.41%; growth 0.40%			x		
67		total 1.36%; employee 1.33%; significant 1.20%; product 1.00%; impact 0.93%; fully 0.90%; operation 0.89%; number 0.86%; gri 0.77%; percentage 0.77%; reporting 0.76%; governance 0.74%; service 0.72%; indicator 0.70%; sustainability 0.69%; performance 0.68%; organization 0.62%; environmental 0.60%; economic 0.59%; body 0.58%					x
68	**Board of directors**	director 4.43%; board 4.42%; committee 2.91%; management 1.72%; member 1.65%; annual 1.62%; financial 1.53%; risk 1.46%; meeting 1.42%; audit 1.41%; executive 1.35%; shareholder 1.26%; governance 1.25%; corporate 1.23%; chairman 0.88%; internal 0.87%; general 0.86%; information 0.83%; control 0.73%; remuneration 0.72%			x		
69	**Stakeholder information**	program 1.91%; community 1.24%; service 0.73%; organisation 0.72%; management 0.68%; performance 0.67%; government 0.65%; work 0.52%; staff 0.48%; people 0.43%; information 0.43%; review 0.43%; reporting 0.40%; financial 0.40%; sydney 0.40%; nsw 0.38%; act 0.38%; total 0.38%; development 0.38%		x			
